# Joint analysis of sequence data and single-nucleotide polymorphism data using pedigree information for imputation and recombination inference

**DOI:** 10.1186/1753-6561-8-S1-S20

**Published:** 2014-06-17

**Authors:** Sunah Song, Robert Shields, Xin Li, Jing Li

**Affiliations:** 1 Department of Electrical Engineering and Computer Science, Case Western Reserve University, Cleveland, OH 44106, USA; 2 Department of Pathology, Stanford University, Stanford, CA 94305, USA

## Abstract

We developed a general framework for family-based imputation using single-nucleotide polymorphism data and sequence data distributed by Genetic Analysis Workshop 18. By using PedIBD, we first inferred haplotypes and inheritance patterns of each family from SNP data. Then new variants in unsequenced family members can be obtained from sequenced relatives through their shared haplotypes. We then compared the results of our method against the imputation results provided by Genetic Analysis Workshop organizers. The results showed that our strategy uncovered more variants for more unsequenced relatives. We also showed that recombination breakpoints inferred by PedIBD have much higher resolution than those inferred from previous studies.

## Background

Next-generation sequencing (NGS) technologies have profoundly changed the landscape of genetic studies [1]. Although the cost of sequencing is becoming more affordable, increasingly more studies are choosing NGS as the primary platform to collect data, either at the whole genome level or for targeted regions. However, costs of sequencing thousands of individuals and the downstream analysis are still prohibitively high. On the other hand, many projects have already accumulated single-nucleotide polymorphism (SNP) data from previous studies. In such cases, researchers only need to sequence a small subset of family members (e.g., proband and parents) to reduce the costs. By jointly analyzing sequence data from a subset of family members together with SNP data from the families, computational approaches may fully recover variant information in unsequenced members. The data distributed by Genetic Analysis Workshop 18 (GAW18) provide an excellent example based on this design strategy. Many of the pedigrees are very large, and all of them have a significant number of members without SNP genotypes, which makes the imputation computationally very challenging. Our laboratory has recently developed an efficient haplotype inference algorithm called PedIBD, which is designed specifically for large pedigrees with many untyped individuals [2]. By taking advantage of haplotypes inferred by PedIBD using SNP data, we developed a procedure to computationally impute variants for unsequenced individuals based on haplotype sharing between them and their sequenced relatives. The advantage of our approach over the imputation provided by GAW lies in the fact that whereas our approach can take each pedigree as a whole when inferring haplotype or inheritance, GAW had to partition big pedigrees into smaller families. Our approach thus will provide more complete and more accurate results. In addition, based on the provided SNP data, we can also provide inferred recombination breakpoints with high resolution within each pedigree.

## Methods

### Data

We focused our analysis on chromosome 3 of the GAW18 dataset. The dataset consists of 1389 individuals from 20 families. Among them, 959 individuals were genotyped using SNP chips. In addition, a subset of 464 genotyped individuals were also sequenced. The total number of SNPs from the chip data is 65,519. Because only rs numbers of these SNPs were provided, we obtained their map positions from the NCBI dbSNP database (Build 37). Nineteen SNPs were removed because they either had no matched rs numbers or the SNPs with the same rs numbers were mapped to a different chromosome. Sequence data was converted to A/C/G/T format using VCFtools [3]. The total number of SNPs called from sequence data is approximately 1.75 million per individual. After removing SNPs with a high missing rate (>5%), the total number of sequence variants that used in our analysis is approximately 1.69 million.

### Analysis

**Figure 1:**
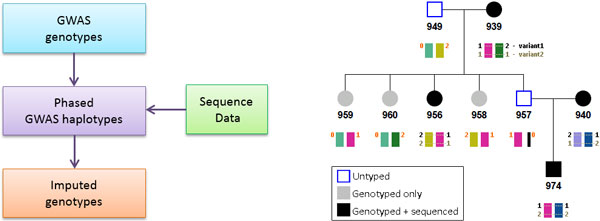
**The imputation framework (left) and an example illustrating the imputation procedure (right)**. In the example, individuals with grey color have single-nucleotide polymorphism (SNP) chip data from genome-wide association studies (GWAS), and individuals with black color have both chip data and sequence data. The haplotypes in this segment are labelled using different colors and they are inferred based on GWAS data. Notice that both haplotypes of individual 949 and one haplotype of individual 957 can be recovered based on the information of their children (the missed haplotype is illustrated using a thin black bar). However, only one haplotype can be recovered for 957 because he only has one child. The two variants are from sequence data (1 and 2 are alleles, and 0 is missing). For the first variant, because member 974 is homozygous genotype (1, 1), the alleles on its two haplotypes (pink and dark blue) can be assigned. Subsequently, the alleles on the light blue haplotype of member 940, the yellow haplotype of member 956, and the green haplotype of member 939 can be resolved (all three have sequence data). For all the other members, their alleles can be imputed based on the color of their haplotypes. However, haplotype light green (in members 949, 959, and 960) cannot be imputed because it has not occurred in any sequenced individual, thus showing missing one allele. For the second variant, our algorithm will identify a conflict because member 974 assigns allele 2 to the pink haplotype, and member 939 assigns allele 1 to the pink haplotype.

Our family-based imputation approach works in several steps. First, recombination breakpoints are inferred and haplotypes are assigned at each recombination-free segment for each individual with SNP chip data using PedIBD. Some individuals without chip data may also be assigned some unique haplotypes based on the inferred inheritance (e.g., untyped parents with typed children). Then, for newly discovered SNPs from sequenced individuals, at each individual locus, the allele on a haplotype can be determined if a sequenced individual sharing the same haplotype is homozygous at this locus. After all homozygous SNPs have been processed, the information can be propagated to heterozygous SNPs if the allele on one haplotype has already been assigned. Genotypes of unsequenced individuals can then be imputed based on their assigned haplotypes (see Figure 1 for the framework and an example). Conflicts may occur when the algorithm tries to assign different allele types to the same haplotype. Conflicts reflect inconsistency between inferred inheritance from chip data and observed SNPs from sequence data. Although there is a possibility that the inferred inheritance could be wrong, a significant majority of conflicts are actually due to high genotype calling errors from sequence data. One should notice that under the assumption that genotyping errors are randomly distributed among all SNPs in sequenced individuals, the total number of loci with conflicts will be proportional to the number of SNPs as well as the number of individuals with genotyping errors even when the typing error rate is a constant. Therefore, the total number of loci with conflicts increases with the size of a pedigree and can thus be substantial in large pedigrees.

**Figure 2:**
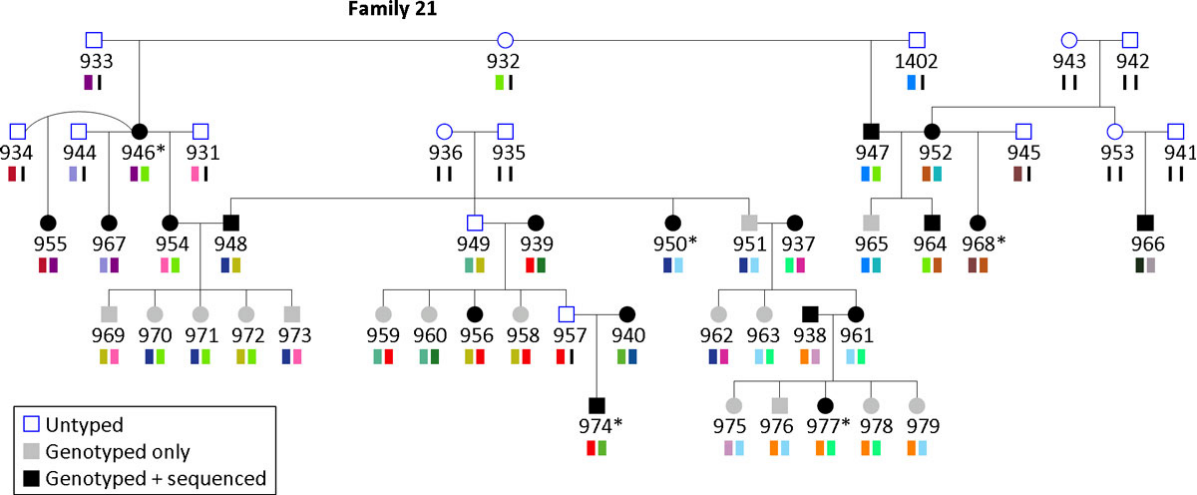
**The pedigree structure of family 21**. The figure shows the pedigree information of family 21 and one haplotype segment inferred by PedIBD for each individual. It also shows which individuals have been imputed and 5 masked individuals who selected for imputation accuracy with asterisks. The legends are the same as those in Figure 1 (right).

Figure 2 shows the pedigree structure of family 21 and one haplotype segment inferred by PedIBD. There are several characteristics of the proposed algorithm. First, because information from the whole pedigree has been used, it is possible that haplotypes for individuals with no data at all can be recovered (e.g., individual 949). It is also possible that only one of the two haplotypes of an individual with no data can be recovered (e.g., individual 957). Second, loci with inconsistent genotypes called from sequence data can be identified (e.g., locus 2 in Figure 1, right). Third, at a variant locus identified from sequence data, if there are no sequenced individuals with homozygous genotypes, the phase at this position cannot be determined.

However, because most new variants from sequence data are rare, the probability of having no homozygous genotypes is extremely low.

For each offspring in a family, a switch on its haplotype assignment indicates a recombination event. We collected all recombination events on chromosome 3 and examined the resolution of recombination breakpoints.

## Results

### Inconsistency between single-nucleotide polymorphism chip data from genome-wide association studies and sequence data

Among 65,500 SNPs from genome-wide association studies (GWAS) data, 63,803 of them were rediscovered from prefiltered whole genome sequencing (WGS) data. Because of high accuracy of chip data, we treated the genotypes from GWAS data as ground truth and first examined the SNP-calling accuracy of WGS data on this subset. Families 14, 15, 23, and 25 were excluded from our analysis because they did not have any sequenced individuals. The inconsistency rate is about 5.50% on average (Table 1). After eliminating families 7, 9, and 11, which had unusual high rates of missing in GWAS data, the inconsistency rate is about 2.25%. We anticipated the issue that the allele types from GWAS data and from sequence data may be encoded differently (i.e., different strands) and did not include discrepancies when alleles are A and T (or G and C). Among the inconsistent genotypes (excluding families 7, 9, and 11), 34.26% were caused by missing genotypes in GWAS, 60.17% were caused by missing in WGS, and the remaining (5.57%) were mismatches. The very high inconsistency for families 7, 9, and 11 was mainly caused by high missing rates of GWAS data in these families. The average missing rate was 2.0% for WGS and 0.72% for GWAS data (excluding families 7, 9, and 11). Both measures indicate that for joint analysis of SNP and sequence data, one should not only impute variants in unsequenced or untyped individuals but also impute these missed or incorrectly called SNPs. For the remaining analysis, we have replaced the incorrect genotypes from sequence data using the genotypes from GWAS data.

**Table 1:** Missing rate and Inconsistency between whole genome sequencing and genome-wide association studies

Family ID	Total number of individuals			Missing rate		Genotype inconsistency between GWAS and WGS			
	
	All	GWAS	WGS	GWAS	WGS	GWAS and WGS	Cause of genotype inconsistency		
				
				65,500	1,697,985	63,803	Missing in GWAS	Missing in WGS	Mismatch
2	107	86	43	1.18%	2.31%	3.20%	67.55%	29.72%	2.73%
3	98	77	38	0.17%	1.66%	0.84%	19.98%	72.31%	7.71%
4	97	64	39	0.17%	2.34%	1.38%	15.80%	73.59%	10.60%
5	91	68	40	0.13%	1.56%	0.82%	20.41%	72.47%	7.12%
6	88	64	39	0.84%	2.06%	2.07%	59.85%	36.38%	3.77%
7	89	36	30	14.59%	1.58%	17.58%	96.60%	2.95%	0.45%
8	84	68	25	3.31%	2.18%	9.23%	89.44%	9.33%	1.23%
9	81	33	27	13.34%	1.50%	16.38%	96.63%	2.90%	0.47%
10	83	64	40	2.42%	1.86%	4.43%	81.79%	16.71%	1.50%
11	76	35	29	20.57%	1.88%	24.81%	96.92%	2.63%	0.45%
16	59	48	26	0.10%	1.62%	0.82%	14.04%	78.04%	7.93%
17	57	42	20	0.30%	2.58%	1.45%	18.22%	75.43%	6.35%
20	51	36	20	0.36%	2.12%	1.43%	28.55%	65.70%	5.76%
21	50	35	19	0.11%	2.49%	1.26%	4.83%	90.21%	4.96%
27	44	35	17	0.16%	2.22%	1.27%	14.98%	77.98%	7.04%
47	27	22	12	0.09%	2.06%	1.04%	9.89%	84.35%	5.76%

	1182	813	464	*3.61%	*2.00%	*5.50%	*45.97%	*49.42%	*4.61%
				^0.72%	^2.08%	^2.25%	^34.26%	^60.17%	^5.57%

Asterisks (*) and Carets (^) indicate averaged numbers across families, with (*) and without (^) families 7, 9, and 11.

### Comparison of imputation results between Genetic Analysis Workshop and our approach

**Figure 3:**
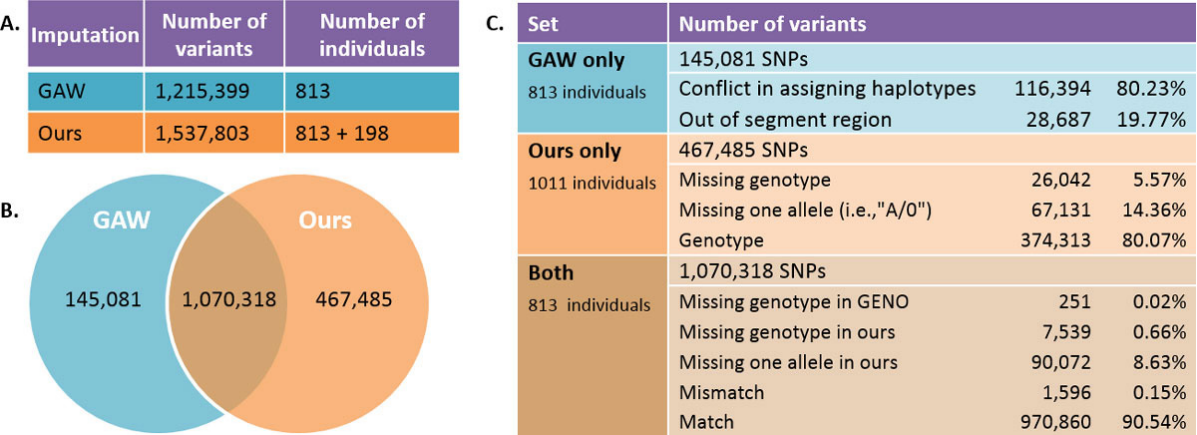
**Imputation results from our approach and data provided by Genetic Analysis Workshop (GAW)**. All numbers are averaged on individual. A) Imputation results of our method and GAW as the number of imputed variants and individuals. B) Overlaps between two sets. C) Itemized comparison between two sets.

We compared our imputation results with the GENO dataset provided by GAW, which recovered 1.2 million variants for 813 individuals (including sequenced individuals themselves). GAW took a 2-step procedure for imputation: a preliminary imputation based on population level information alone in the first step and an additional imputation procedure using pedigree information in the second step using SimWalk2 and Merlin [4,5]. Neither program can handle pedigrees as large as the GAW18 families, so both required large families to be partitioned into smaller subfamilies. In contrast, by taking each pedigree as a whole, our method was able to recover approximately 1.53 million SNPs for 1011 individuals (these include an additional 198 individuals without sequence data or GWAS data), which accounts for 90.6% of total 1.69 million variants. Both the number of imputed SNPs and the number of imputed individuals by our approach are substantially higher than those given by the GAW. For the 9.4% remaining variants, our imputation method found that 7.39% had conflicts that are similar to the one in Figure 1 (right, second locus), caused by calling errors from sequence data. About 2% of them were located between haplotype segments. For the rest 0.01%, all of the sequenced individuals in each pedigree were heterozygous; therefore, genotypes of unsequenced individuals cannot be imputed. Among 1,070,318 common variants imputed by both methods for the 813 individuals, we found 0.15% of genotype mismatch between 2 sets. Our approach has imputed 467,485 more variants than the GENO dataset but missed 145,081 SNPs. The majority of the missed SNPs (>80%) are due to conflicts discovered by our program. This is consistent with the genotype-calling error rate from sequence data. The remaining SNPs were missed because their positions were out of haplotype segment regions. The summary of results can be found in Figure 3.

### Imputation accuracy

We further evaluated the imputation accuracy by assuming sequence data of some individuals were unknown [6]. We selected 5 individuals from family 21 (Figure 2 and Table 2). For each of them, we masked all of their genotypes (i.e., all genotypes were set to be "missing"), performed the imputation procedure, and then assessed the imputation accuracy as the proportion of correctly imputed alleles. Table 2 shows the pedigree relationship information for masked individuals and imputation accuracy. Each individual represents a distinct relationship within the family. Results show that imputation on individual 946 has the highest accuracy. Four of her close relatives (three children and one half-sibling) have been sequenced, and the average missing rate of the sequenced relatives was 2.13%. Most of her genotypes can be inferred because even if there is a missing variant in one of sequenced relatives at a locus, the other relatives may provide enough information to derive her genotypes. Individual 977 has the lowest accuracy, although both parents have been sequenced. Theoretically, one should be able to infer a child's genotypes from both parents if the inheritance is given. However, in this case, not only does she have a smaller number of sequenced relatives, but the missing rate of the father (4.17%) is also much higher, both of which contribute to the low accuracy.

**Table 2:** Pedigree information of masked individual and imputation accuracy.

Masked individual ID	Pedigree information				Accuracy (%)
		
	First-degree relationship			Second-degree relationship	
		
	Parents	Children	Siblings	Half-sibling, grandparent, grandchildren, aunt and uncle, niece and nephew	
**946**	2(U)	3(GS)	0	5(G) + 1(GS)	99.43
**950**	2(U)	0	1(U) +1(G)+1(GS)	10(G) + 2(GS)	98.33
**968**	1(U)+1(GS)	0	0	3(U) + 1(G) + 1(GS)	96.33
**974**	1(U)+1(GS)	0	0	1(U) + 3(G) + 2(GS)	95.72
**977**	2(GS)	0	4(G)	3(G) + 1(GS)	91.37

*G*, genotyped only, *GS*, genotyped + sequenced; *U*, untyped.

### Recombination breakpoints

The haplotypes and recombination breakpoints have been obtained from all families based only on GWAS data. Overall, there are a total of 3089 recombination events identified. Among them, a fraction still cannot be determined from their parental sources because of missing genotypes in parents. After filtering out recombination events with unknown parental origins, our final dataset had 1361 maternal and 933 paternal recombination events. Because of homozygous genotypes, recombination breakpoints cannot always be within two adjacent SNPs. Still, the resolution of our inferred recombination breakpoints is very high, with more than 94% of them within 20-kb range, and the median length is about 8 kb, which is a great improvement over previous results [7,8].

## Discussion

In this study, we have proposed a computational framework to infer haplotypes and recombination breakpoints and finally impute genotypes based on a subset of sequenced members in a pedigree. Results on GAW18 data have demonstrated that (a) our approach is efficient for extremely large pedigrees and (b) we imputed more variants and more individuals than the one provided by GAW organizers.

Our approach can be further improved in several directions. First, data quality, including missing and genotyping errors, can have a substantial effect on the final results. Many genotyping errors are actually Mendelian consistent, which makes error detection a challenging task. With the development of sequencing technologies as well as SNP calling algorithms, we expect the quality of genotyping calling from sequence data will improve, which in turn will improve our imputation results (e.g., reduce the number of conflicting loci). Second, given the high density of SNPs, population-level linkage disequilibrium can be used in imputation even for family data. Investigating approaches that can jointly consider information within families and information at the population level will be our future work. Third, our haplotype segments are defined based on all observed recombination events in a family. Therefore, the haplotype segments of a particular individual may have been cut short unnecessarily from recombination breakpoints of other individuals, resulting in some variants between haplotype segments being dropped. We will define haplotype segmentations of each individual based on her or his own recombination breakpoints, which will reduce the number of dropped variants. Last, our results show that the strategy of sequencing only a small subset of family members and imputing others is very effective. However, the final imputation results may depend on many factors, such as number and type of relationships of sequenced relatives, as well as the quality (e.g., missing rate) of sequence data. A truly important decision is how researchers select individuals to sequence to optimize the amount of information acquired within the constraints of a budget.

## Competing interests

The authors declare that they have no competing interests.

## Authors' contributions

SS, SR, and LJ designed the study. SS conducted imputation analysis, and SR performed the recombination study. LX contributed to analysis of the data. SS, SR, and LJ drafted the manuscript. All authors read and approved the final manuscript.
